# Sensory Classification of Brazilian *Coffea arabica* by Laser-Assisted Rapid Evaporative Ionization Mass Spectrometry and Machine Learning Algorithms

**DOI:** 10.1021/acsomega.5c00404

**Published:** 2025-04-29

**Authors:** Victor Gustavo Kelis Cardoso, Julia Balog, Guilherme Post Sabin, Leandro Wang Hantao

**Affiliations:** † Universidade Estadual de Campinas, Instituto de Química, Rua Monteiro Lobato 270, Campinas, SP 13083-862, Brasil; ‡ Instituto Nacional de Ciência e Tecnologia em Bioanalítica (INCTBio), Rua Monteiro Lobato 270, Campinas, SP 13083-862, Brasil; § Waters Research Center, Záhony utca 7, Graphisoft Park, Budapest 1031, Hungary; ∥ OpenScience, Rua Conceição 233, Campinas 13010-050, Brasil

## Abstract

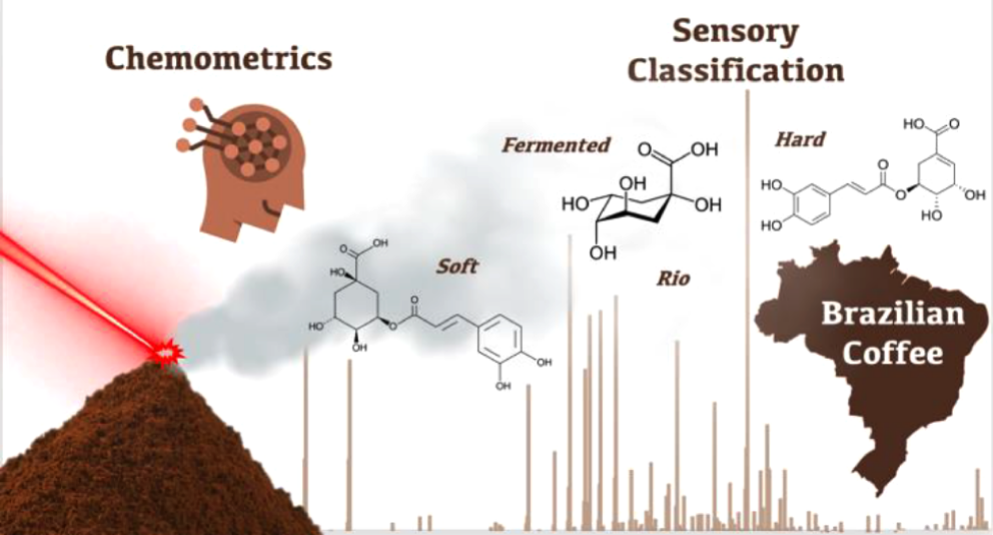

Brazil plays an important role in coffee quality assessment since it is the top producer and exporter. New technologies must be developed to increase production and ensure product quality. Thus, this study presents an application of laser-assisted rapid evaporative ionization mass spectrometry (LA-REIMS) to fingerprint more than 800 Arabica coffee samples. These samples were divided into six sensory classes by professional tasters according to the Brazilian official classification. Machine learning algorithms were applied for a better understanding of complex fingerprints, and their performances were compared. Partial least-squares discriminant analysis (PLS-DA) was inferior in its predictive capability compared to support vector machines (SVM) and artificial neural networks (ANN), which achieved up to 100% accuracy. The high sensitivity to distinct sensory classes enabled a tentative identification of spectral signals, such as fatty acids, chlorogenic acids, and phospholipids, which are likely being related to these properties in Arabica coffee for the first time.

## 1 Introduction

Coffee is a popular beverage that attracts numerous consumers worldwide due to its stimulating properties and pleasant flavor and taste.^1^ This beverage is obtained from the fruits of plants belonging to the *Coffea* genus.^2,3^ Among more than a hundred species in this genus, *Coffea arabica* and *Coffea canephora* are the most notable. *C. arabica*, popularly known as Arabica coffee, accounts for the majority of coffee production due to its superior sensory properties, such as higher acidity, lower bitterness and caffeine content, and a more intense aroma.^4,5^ As a result of its popularity, coffee also holds significant economic importance, with global trade reaching approximately 36 billion dollars in 2021. Brazil is the world’s largest coffee producer, responsible for 17% of overall coffee trade and 28% of nonroasted coffee exports in 2021.^6^ In that year, 39.4 million coffee burlaps (60 kg each) were exported.

As the leader in coffee production and exportation, Brazil plays a crucial role in regulating and ensuring the quality of coffee for fair pricing and reliability in exports. The Brazilian Ministry of Agriculture, Livestock, and Food Supply established Normative Instruction No. 8 on June 11, 2003, to regulate and classify nonroasted coffee beans,^7^ a system known as the Brazilian official classification. This regulation considers several parameters, such as defective beans, impurities, grain sizes, color, moisture, and sensory perception based on the cupping test.^7,8^

Regarding the sensory classification of Arabica coffee, it is categorized into *strictly soft*, *soft*, *barely soft*, *hard*, *rioysh*, *rio*, and *riozona*, in descending order of quality.^8,9^ The first four classes are considered high-quality fine coffee, whereas *rioysh*, *rio*, and *riozona* are classified as phenicated beverages due to flavors resembling phenol or iodoform. Other unofficial sensory profiles may also occur, such as fermented or immature, resulting from unwanted microbial fermentation or premature harvest, respectively.^10^

Sensory analysis is the most subjective parameter defined in normative regulations due to the lack of well-defined standards for an accurate assessment, relying exclusively on professional tasters’ judgment.^11^ Despite extensive training procedures for sensory panels, studies show that these professionals are subject to significant deviations.^11,12^ Thus, new methods must be developed to improve the understanding of coffee sensory profiles and assist professionals in making accurate decisions. Additionally, rapid approaches are highly desirable to meet the growing demand of the coffee market.

Instrumental techniques have been successfully employed as alternatives to classical methods^13^ and could serve as valuable tools for coffee sensory classification. Various methods have been reported in recent years for analyzing coffee using such techniques. However, only a few studies have focused on understanding the sensory profiles of *C. arabica* based on the Brazilian official classification, primarily employing vibrational spectroscopy,^9,14,15^ gas chromatography,^16^ and mass spectrometry (MS).

Nevertheless, these techniques present certain limitations, such as low sensitivity, lack of compound-level information, slow results, or, in the case of mass spectrometry, extensive sample preparation steps. These challenges can be effectively addressed with recent advancements in ambient ionization mass spectrometry,^17^ which allows real-time results with minimal sample preparation or manipulation.^18,19^

This research highlights laser-assisted rapid evaporative ionization mass spectrometry (LA-REIMS), an automated system that enables sample vaporization via laser ablation, followed by aspiration into the spectrometer ion source.^20,21^ The potential of LA-REIMS fingerprints for coffee analysis was demonstrated in a proof-of-concept study by our group, achieving accuracies of up to 96% in predicting acidity, bitterness, body, and other properties of espresso coffee capsules.^22^ Therefore, analyzing real samples of Brazilian *C. arabica* using LA-REIMS could provide valuable insights into sensory profiles, enhance predictive model training, and support scalability for large-scale applications. The feasibility of such MS-based techniques for coffee analysis has been increasing the recent years for numerous purposes, such as authentication^23,24^ and geographic classification.^25,26^

Understanding the data provided by LA-REIMS systems is challenging due to the numerous *m*/*z* signals generated by a high-resolution mass spectrometer and the rapid measurement process.^22,27^ The large volume of data produced during a harvest period, for example, could be impractical for manual interpretation, necessitating advanced approaches to obtain reliable outcomes from spectral fingerprints.^22,23,25,27^ To address this issue, coffee samples are divided into sensory classes, making discriminant algorithms the most suitable approach for predictions on a categorical scale.

Partial least-squares discriminant analysis (PLS-DA) is the most widely used method for discriminant purposes, as it aims to project data into a lower-dimensional space while maximizing the covariance between data and classes.^27,28^ However, PLS-DA has limitations when dealing with heterogeneous, extensive, and complex data sets. Algorithms such as support vector machines (SVM) and artificial neural networks (ANN) have proven to be superior to PLS-DA in certain scenarios due to their ability to handle overlapping classes, perform nonlinear modeling, and achieve higher generalization capabilities.^29,30^ These algorithms have recently gained popularity, partly due to advancements in training methodologies such as Bayesian optimization, which enables automated, fast, and efficient selection of modeling parameters.^30,31^

This study demonstrates the effectiveness of LA-REIMS combined with machine learning algorithms as a powerful tool for the sensory classification of Brazilian *C. arabica*. By leveraging high-resolution mass spectrometry fingerprints, the proposed approach successfully differentiated sensory classes with high accuracy, achieving up to 100% classification performance in select categories. The identification of potential sensory markers, including fatty acids and phospholipids, represents a significant advancement in coffee quality assessment, offering new insights into the chemical composition associated with distinct sensory attributes. Future research should focus on expanding spectral libraries and refining predictive models to enhance the resolution of intermediate sensory classes. By bridging analytical chemistry and artificial intelligence, this work paves the way for more precise, efficient, and reproducible coffee classification methods, ultimately benefiting producers, regulatory agencies, and consumers alike.

## 2 Materials and Methods

### 2.1 Samples and Chemicals

**Figure 1:**
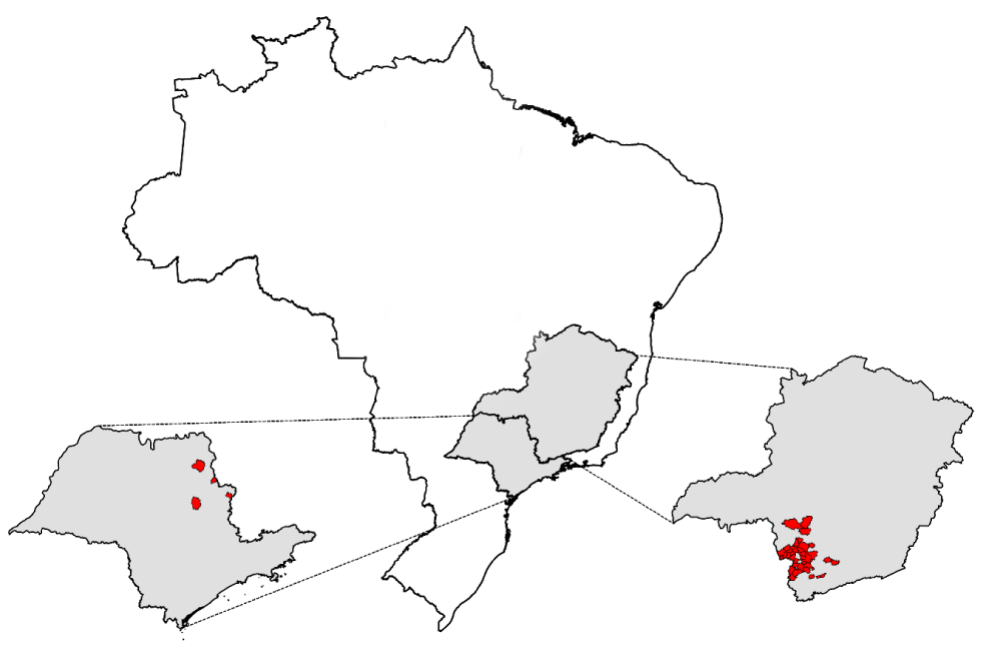
Geographical location of *C. arabica* samples used in the study.

This study analyzed 824 samples of Brazilian *C. arabica* harvested in 2020 from various farms in cities across the São Paulo and Minas Gerais states (Figure 1). The samples were provided by a coffee cooperative based in Minas Gerais, Brazil. The coffee beans were roasted to 85 points on the Agtron scale (light roast), ground, and stores in microtubes. Five different portions of those roasted and ground coffee were evaluated by two panelists using the Brazilian official classification for sensory analysis. Each coffee sample was then categorized into six sensory groups considering the five cups, as detailed in Table 1. All these steps were carried out by the coffee cooperative, which also provided the sensory evaluation results. The microtubes containing the coffee samples were stored at -20 °C until analytical procedures were performed.

**Table 1:** Sensory Scale and Description of Different Classes of *C. Arabica*

Class	Number of samples	Overall quality	Sensory description
*Barely Soft*	129	Excellent	Low acidity, pleasant aroma, and sweet and smooth taste.
*Hard +*	139	Good	Hard coffee, but subtly superior taste and aroma related to barely soft in one or two cups.
*Hard*	140	Regular	Acrid, astringent, and harsh taste, lack of sweetness and softness, but without unpleasant sensations.
*Hard –*	139	Bad	Hard coffee, but mild iodoform taste related to rio in one or two cups.
*Rio*	138	Very bad	Accentuated iodoform, phenolic, pungent or medicinal taste.
*Fermented*	139	Regular	Vinegar taste resulted by undesired fermentation.

Ultrapure water (Type 1) was obtained from a Milli-Q purifier (Millipore, USA). Isopropanol Chromasolv MS grade was purchased from Merck KGaA (Germany), and the leucine-enkephalin standard was obtained from Waters Corporation (USA).

### 2.2 Instrumentation and Analysis

Coffee samples were analyzed using a SYNAPT XS hybrid time-of-flight mass spectrometer (Waters Corporation, USA) in tandem with a REIMS ionization source in the negative ionization mode, coupled to an automated laser-ablation sampler prototype developed by the Waters Research Center (Hungary). A continuous flow of leucine-enkephalin in isopropanol (100 μg L^-1^) at a flow rate of 150 μL min^-1^ was used to promote analyte ionization in the sample vapor and perform lock mass calibration. The cone voltage and heater bias were set to 30 V. Spectral information was acquired within the *m*/*z* range of 50–1200 and processed using MassLynx V4.2 software (Waters Corporation, USA). A standard solution of sodium formate (Waters Corporation, USA) was used for the calibration of the mass spectrometer.

Aliquots of 1.00 ± 0.05 g of coffee powder were placed in microcentrifuge tubes, followed by the addition of 1 mL of Type I water. The tubes were then homogenized using a vortex mixer for 1 min. Each moist coffee powder sample was transferred to a well plate for measurement. Each sample underwent laser ablation with 20 shots in 1 s, corresponding to a sampling frequency of 20 Hz, and the generated vapor was aspirated into the mass spectrometer for spectral acquisition. Each sample was analyzed in six sequential technical replicates in different regions of the same sample, totaling 4,944 measurements. Spectral preprocessing was performed using Progenesis Bridge v1.0.29. The raw files were converted to “*.mzXML” format using ProteoWizard for further chemometric analysis.

### 2.3 Data Preprocessing

Mass spectrum files were imported into MATLAB version 9.11 R2021b with the Bioinformatics Toolbox version 4.15.2 (MathWorks, USA). The 4,944 mass spectra (rows) were binned into 100,000 mass bins (columns), generating a data matrix of these dimensions. The data matrix was normalized by length, and the sample average spectrum was calculated to address concerns about sample heterogeneity and measurement representativity, reducing the data set to 824 mass spectra.

Noise filtering was performed by removing signals with intensities lower than 0.20% of the maximum intensity, reducing the number of *m*/*z* features to 3,473. The data set was randomly divided into training (700 samples, 85%) and validation (124 samples, 15%) sets. These sets were separately Pareto-scaled to reduce signal magnitude (mask effect) without increasing noise and deviations. Mean centering was applied only for PLS-DA models.

PLS-DA, ANN, and SVM were used to train classification models for coffee samples, categorizing them into six sensory classes: *barely soft*, *hard+*, *hard*, *hard–*, *rio*, and *fermented*.

### 2.4 Partial Least-Squares Discriminant Analysis (PLS-DA)

PLS_Toolbox 9.0 (eigenvector, USA) was used to develop the PLS-DA models. Latent variables (LVs) were selected based on the lowest Venetian-blind cross-validation average errors. Samples with high Hotelling T^2^ and Q residual values were identified as outliers and subsequently removed from the model.

### 2.5 Support Vector Machine (SVM)

SVM models were trained using the function “*fitcsvm*” available in Statistics and Machine Learning Toolbox version 12.2 (MathWorks, USA). A linear kernel function was applied for data transformation. Bayesian optimization (default mode) was used for the automated selection of model parameters, including box constraint and kernel scale. The expected improvement plus acquisition function was employed in Bayesian optimization with 100 iterations to achieve the optimal models, considering the 5-fold cross validation.

### 2.6 Artificial Neural Networks (ANN)

ANN models were trained using the function “*fitcnet*” Statistics and Machine Learning Toolbox version 12.2 (MathWorks, USA). The input layer contained 3,473 neurons, representing the initial data fed into the model. The optimal parameter combination was determined through Bayesian optimization (default mode), which was used to identify the most suitable activation function, lambda values, number of layers and their sizes, and the application of standardization. The expected improvement plus acquisition function was applied in Bayesian optimization with 50 iterations to achieve the best models, considering the 5-fold cross validation.

The limited-memory Broyden-Fletcher-Goldfarb-Shanno quasi-Newton algorithm (LBFGS) was employed to optimize weights and biases through the backpropagation algorithm. SoftMax was used as the activation function for the output layer.

## 3 Results and Discussion

### 3.1 Coffee Analysis by Laser-Assisted Rapid Evaporative Ionization Mass Spectrometry

**Figure 2:**
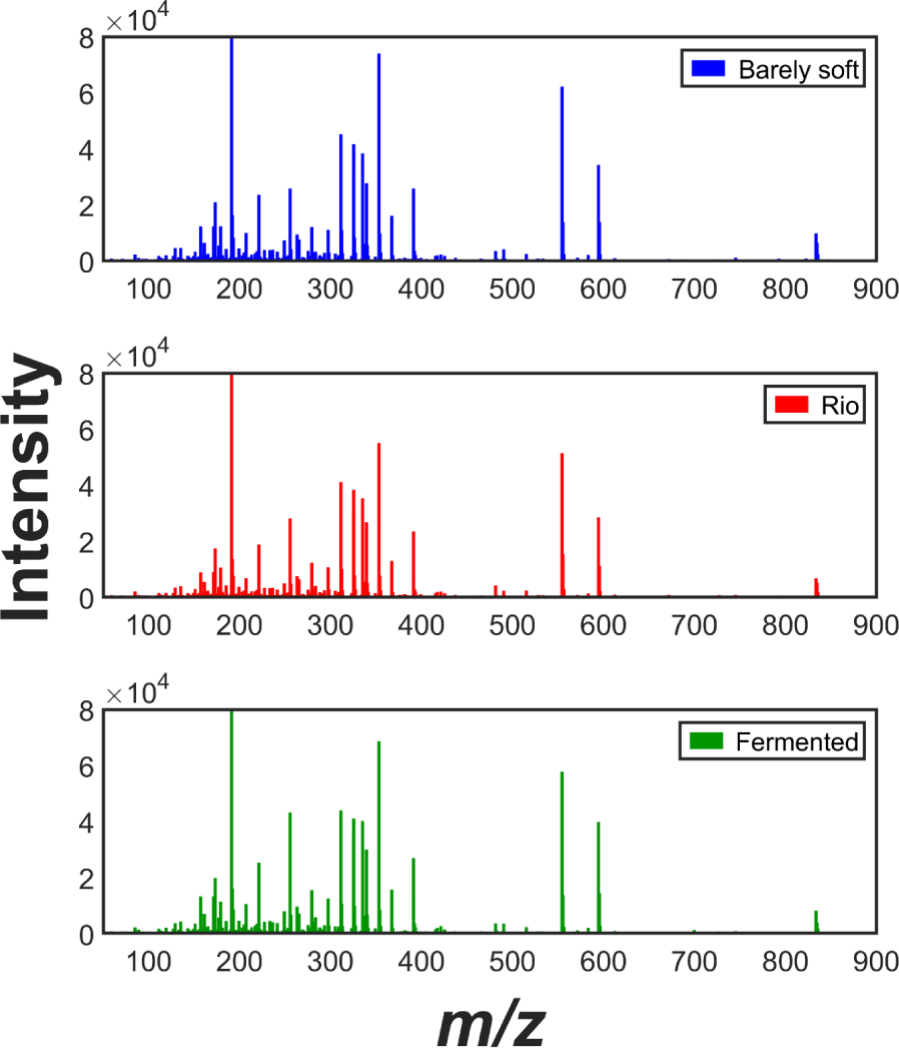
Average mass spectra obtained by LA-REIMS for *C. arabica* samples from the most distinct sensory classes.

Coffee analysis using LA-REIMS has proven to be an useful technique in a previous study conducted by our group^22^ in terms of speed, ease of use, high throughput, and scalability. Various sensory properties were accurately predicted based on the reproducible spectral fingerprints obtained through this method. A similar approach was applied to the current coffee samples, yielding suitable spectral fingerprints (Figure 2) with a measured resolution of 26,000 at the leucine-enkephalin standard peak (*m*/*z* 554.2619). However, some limitations were observed in the present samples, likely related to sample heterogeneity and the granulometry of the coffee powder. The espresso coffee powder used in the previous study consisted of 95% of its particles within the range of 300–600 μm, whereas the current samples have 74% of their particles larger than 840 μm. This difference in granulometry may have led to variations in laser incidence on individual particles and focus-related issues. To address this, six sampling points per sample were averaged to improve representativity.

LA-REIMS has already showed many compounds in coffee in research by us,^22^ which matched with previous investigations by using MS-techniques.^5,32^ Compound assignment is not the aim of this work, but the signals related to chlorogenic acids, fatty acids, and phospholipids were probably observed in the spectral profile, which will be discussed in next sections. Some subtle differences in sensory classes can be identified in Figure 2, but the spectral profile is complex and difficult to be directly related to the properties. Furthermore, interaction among compounds can affect differently the coffee sensory perception. Thus, a fingerprint approach by using multivariate analysis algorithms was selected as the most suitable to deal with the MS coffee data. It allows the achievement of accurate predictive models by using PLS-DA, ANN, and SVM.

### 3.2 Cupping Estimation by LA-REIMS and Machine Learning Algorithms

PLS–DA, SVM, and ANN were applied to the coffee data set to classify samples into their respective sensory categories. Six different classes were considered in this classification: *barely soft*, *hard+*, *hard*, *hard–*, *rio*, and *fermented*. The analyses were conducted on approximately one gram of roasted coffee powder obtained from various burlaps, which may raise concerns regarding the representativeness of the sampling process, reference method, or chemical information due to the limited sample quantity. Nevertheless, it is remarkable that even with a small amount of coffee, it is possible to achieve insights into a larger population encompassing tens or hundreds of coffee burlaps, as presented in the paragraphs below.

PLS-DA used 45 latent variables (LVs) to train the model, which can be justified by the presence of numerous noncorrelated *m*/*z* signals (3,473 *m*/*z* bins), a large sample set with high biological variability (824 samples), and heterogeneous reference values due to the subjectivity of sensory analysis. Despite these challenges, the models were extensively validated, achieving an optimal balance between underfitting and overfitting. PLS-DA attained an accuracy of 61% in predicting the validation set, which, while not exceptionally high, is reasonable given the limitations of the reference values. This suggests that further investigation could enhance classification performance.

**Figure 3:**
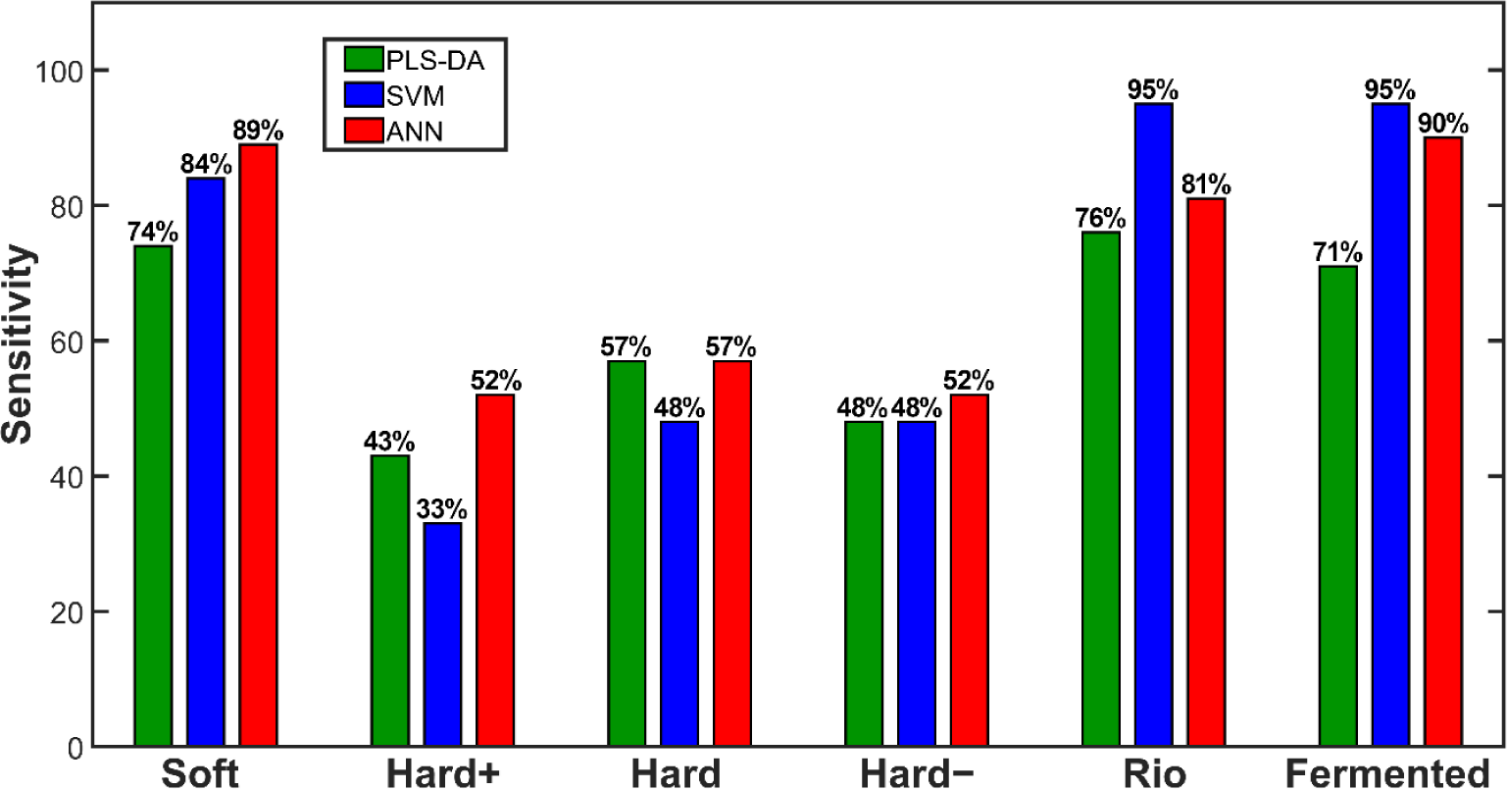
Sensitivity values for each sensory class modeled using PLS-DA, SVM, and ANN.

Additionally, sensitivity values for individual classes indicate that *barely soft*, *rio*, and *fermented* coffees were more easily discriminated due to their higher sensitivity scores, whereas *hard+*, *hard*, and *hard–* presented lower values (Figure 3). This result suggests that intermediate classes do not exhibit distinct markers, as they fall within a gradient between excellent and poor-quality coffees, which could explain their lower sensitivity. This statement is also supported by confusion chart for the model in Figure S3. Moreover, the subtle sensory differences between these intermediate classes may influence the reference values provided by the sensory panel.

An important limitation of PLS-DA was its inability to provide reliable estimations for samples with high Hotelling T^2^ and Q residuals, a common issue in complex sample matrices such as coffee. This limitation particularly affected the classification of fermented coffees, with approximately 20% of samples in this class identified as outliers due to high residuals. Additional information on outliers is provided in Figure S2.

**Figure 4:**
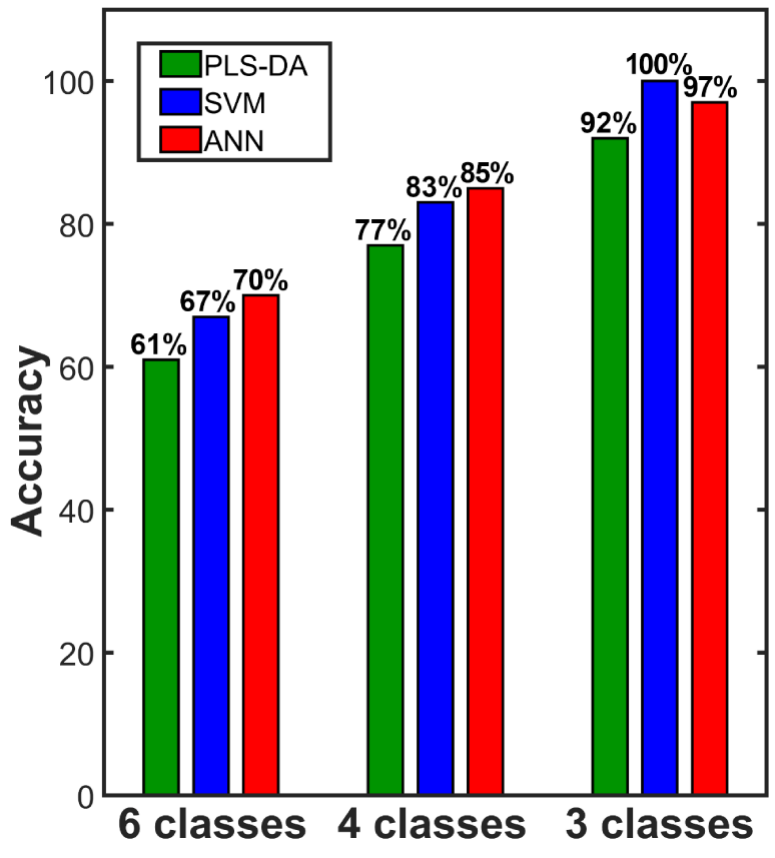
Accuracy values of PLS-DA, SVM, and ANN models when classifying 6, 4, and 3 sensory classes.

Due to the challenges of handling heterogeneous data and the limitations of the reference method, alternative algorithms were applied to train classification models for this complex data set. SVM and ANN were used as alternatives to PLS-DA when dealing with difficult discrimination problems such as this one.^30,33,34^ These algorithms achieved accuracy rates of 67% and 70% for SVM and ANN, respectively, when predicting the validation sets. This represents an improvement of up to 9 percentage points compared to PLS-DA (Figure 4).

However, the most notable increase was observed in well-defined classes, with sensitivity values rising by up to 24 percentage points for *barely soft*, *rio*, and *fermented* coffees (Figure 3). These findings highlight the inherent difficulty of classifying intermediate classes, reinforcing the previously mentioned hypothesis. When the *hard+* and *hard–* classes, which exhibit low sensitivity, were excluded from the spectral library, accuracy values further improved (Figure 4). Moreover, when evaluating only the *barely soft*, *rio*, and *fermented* classes, accuracy levels reached up to 100% (Figure 4).^35^Supporting Information discloses additional figures of merit (Table S2) and confusion charts for the models (Figures S3–S5).

Compared to previous studies, only a few have presented predictive models for the classification of coffee according to the Brazilian official classification. Rezende and colleagues used electrospray ionization mass spectrometry for an exploratory analysis of the sensory profile of *C. arabica*, but they did not describe a validated predictive model. Franca and collaborators, for example, analyzed *C. arabica* using mid-infrared spectroscopy and developed PLS-DA models to classify samples into *soft*, *hard*, *rioysh*, *rio*, and *riozona*, achieving up to 100% sensitivity and specificity for some classes.^9^ Similarly, Azevedo and coworkers applied near-infrared spectroscopy, obtaining up to 92% accuracy in discriminating *soft*, *hard*, *rioysh*, and *rio* coffees.^14^ Ferreira and collaborators used near-infrared spectroscopy and gas chromatography to analyze *C. arabica* and predict its sensory classes on a quantitative scale, generating reasonable models with consistent predictions.

Despite these positive outcomes, it is important to note that all these studies used relatively small sample sizes (fewer than 100 samples). This limited sample size may introduce potential challenges, such as low biological variability or an increased risk of sampling bias, both of which could affect the accuracy of the predicted results. Other studies have also employed predictive models for different sensory classifications of *C. arabica*, but these primarily focused on evaluating blends,^36^ different roasting degrees,^37^ or geographic origins.^38^ However, the differing objectives of these studies make direct comparisons with our results difficult.

Overall, the present study contributes to a deeper understanding of coffee sensory quality by integrating MS-based techniques with advanced data analysis methods. Recent studies have demonstrated the potential of direct MS techniques for coffee classification, such as the work by Yu, Yang, and coworkers, who achieved 99.8% accuracy in predicting coffee origin countries using machine learning methods.^25^ Similarly, Chen, Tsai, and coworkers applied direct analysis MS to authenticate palm civet-digested coffee beans, obtaining 99.6% accuracy. Beyond direct MS, recent approaches have combined gas chromatography and MS to classify coffees from different countries,^23^ investigate sensory defects,^39^ and estimate sensory traits.^40^

Despite the lower performance observed for intermediate sensory classes, this reflects the higher complexity of distinguishing samples with similar species, varietal, roasting process, and region — a more challenging task than differentiating coffees from different countries or production systems. The large sample set analyzed here, covering a wide biological variability, enhances the robustness of the proposed models. Moreover, the minimal sample preparation and high-throughput capacity of the method allow the analysis of hundreds of samples per day, making this approach highly suitable for routine quality control. Increasing the number of training samples further improved model performance (results not shown), suggesting that expanding the spectral library could enhance the discrimination of intermediate sensory classes in future studies.

### 3.3 Identification of Possible Markers of Different Sensory Types of Arabica Coffee

**Figure 5:**
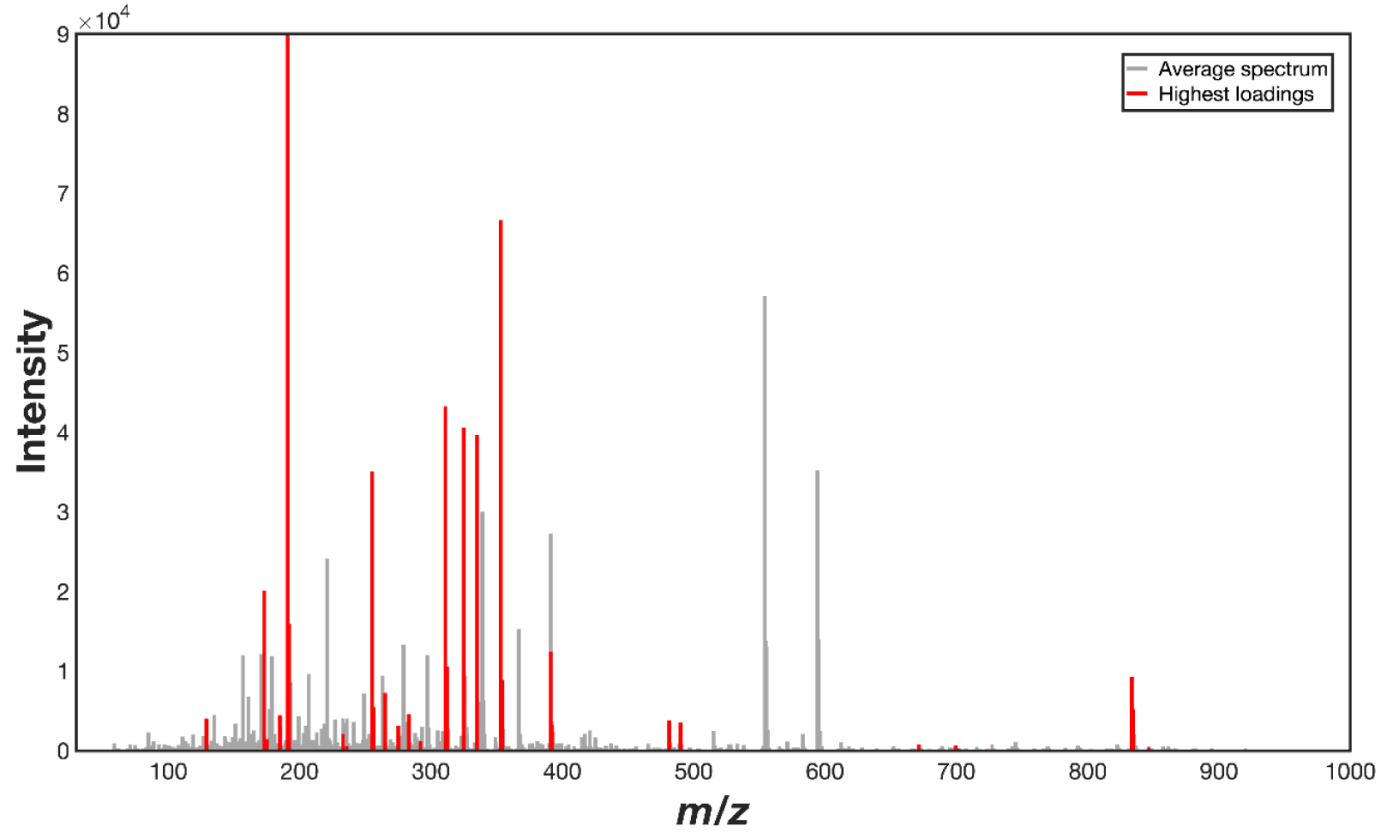
Representative mass spectrum highlighting in red the signals with the highest loadings obtained from the orthogonalization of binary PLS-DA models.

Table 2 provides a tentative identification of possible markers for distinguishing between different sensory types of *C. arabica*. This identification process involved selecting signals with high loading values during the orthogonalization of binary PLS-DA models (one-versus-all). The identified signals or markers are also highlighted in Figure 5. Among the compounds suggested in Table 2 as key markers of *C. arabica* sensory types, chlorogenic acids and their fragments, fatty acids, and phospholipids stand out, aligning with extensive descriptions of coffee composition obtained through mass spectrometry.^5,32,41−45^ Further investigation is required to confirm these markers with greater confidence,^46^ employing complementary techniques such as fragmentation pattern analysis and isomer resolution.

**Table 2:** Signals with the Highest Loading Values on Discrimination of Different Properties in Coffee

**Experimental***m*/*z*	**Mass error**	**DBE**^a^	**Empirical formula [M – H]^-^**	**Proposed structure**	**ID level**(46)	**Property**	**Reference**
129.0186	-5.6	3.5	C_5_H_5_O_4_	Citraconic acid	IV	*Barely Soft*	(37)
173.0452	-2.2	3.5	C_7_H_9_O_5_	-	V	*Barely Soft*	-
175.0174	-	-	-	-	-	*Rio*	-
185.0503	-	-	-	-	-	*Rio*	-
191.0559	-1.2	2.5	C_7_H_11_O_6_	Quinic acid	IV	*Fermented, Rio*	(5,27,34,38,40)
233.0689	9.6	3.5	C_9_H_13_O_7_	-	V	*Rio*	-
236.0593	-	-	-	-	-	*Barely Soft*	-
255.2331	0.5	1.5	C_16_H_31_O_2_	Palmitic acid	IV	*Fermented*	(38)
265.1483	-	-	-	-	-	*Barely Soft*	-
275.1123	-4.7	3.5	C_12_H_19_O_7_	-	V	*Barely Soft*	-
283.2640	-0.8	6.5	C_18_H_25_O_2_	Stearic acid	IV	*Fermented*	(5,27,38)
292.0845	-	-	-	-	V	*Rio*	-
311.1687	-7.9	0.5	C_13_H_27_O_8_	-	IV	*Rio*	-
311.2955	-0.2	1.5	C_20_H_39_O_2_	Arachidic acid	IV	*Fermented*	(27,28)
325.1842	-8.1	0.5	C_14_H_29_O_8_	-	V	*Rio*	-
335.0774	0.4	9.5	C_16_H_15_O_8_	Caffeoylshikimic acid	IV	*Rio*	(5,27)
353.0879	0.3	8.5	C_16_H_17_O_9_	Caffeoylquinic acid	IV	*Barely Soft*	(5,27,34,40)
391.0440	-4.9	16.5	C_21_H_11_O_8_	-	IV	*Fermented*	-
481.2443	0.0	7.5	C_25_H_37_O_9_	Atractyloside II	IV	*Rio*	(5,27,38)
490.1366	-	-	-	-	-	*Barely Soft*	-
671.4639	-2.8	4.5	C_37_H_68_O_8_P	Phosphatidic acid 34:2 (16:0/18:2)	IV	*Rio*	(36)
699.4957	-1.9	4.5	C_39_H_72_O_8_P	Phosphatidic acid 36:2 (18:0/18:2)	IV	*Fermented*	(36)
723.4957	-1.8	6.5	C_41_H_72_O_8_P	Unknown phospholipid	IV	*Fermented*	(36)
725.5144	2.3	5.5	C_41_H_74_O_8_P	Unknown phospholipid	IV	*Fermented*	(36)
833.5164	-2.6	5.5	C_43_H_78_O_13_P	Phosphatidylinositol 34:2 (16:0/18:2)	IV	*Barely Soft*	(36)
846.5272	0.9	6.0	C_44_H_79_O_13_P	Unknown phospholipid	IV	*Barely Soft*	-

a DBE: Double bond equivalent.

The tentative identification of certain signals in Table 2 was not straightforward due to mass errors exceeding 10 ppm, which may be attributed to the compositional constraints applied in this study.

Chlorogenic acids are widely recognized as important compounds in sensory investigations,^41,47^ and similar findings were observed in this study, as a higher intensity of caffeoylquinic acids (CQAs) was detected in *barely soft* coffees. Among the CQAs, 5-caffeoylquinic acid (5-CQA) was previously identified as a marker of low-quality and *rio* off-flavor coffees,^48^ whereas 3-caffeoylquinic acid (3-CQA) has been associated with high-quality coffees.^42^ However, other researchers suggest that this relationship is not straightforward. Their study, which analyzed samples from different origins, cultivars, and postharvest processes, found coffees with high levels of 5-CQA and CQAs in general that exhibited excellent cup quality, and vice versa.

On the other hand, caffeoylshikimic acids and/or caffeoyl-quinides were associated with *rio* coffee. To date, caffeoylshikimic acid and caffeoyl-quinides have not been described as markers of sensory classes in *C. arabica*. However, caffeoyl-quinides have been reported as compounds that contribute to bitterness in coffee, a characteristic undesirable in high-quality coffees.^49^

Quinic acid and its isotopic contributions, which are fragments of chlorogenic acids, present inconclusive findings, as they appear to be important across all classes, likely due to fluctuations in their intensity. However, peaks corresponding to leucine-enkephalin are likely associated with a reduction in the base peak (quinic acid) and are specifically related to *rio* coffee, along with its isotopic contributions. Quinic acid is extensively described in the literature as a compound contributing to bitterness, sourness, and astringency in coffee. However, further studies are needed to better understand its role in the sensory classification of *C. arabica*.

Overall, fatty acids played a significant role in *fermented* samples, where high intensities of palmitic, stearic, and arachidic acids were observed. These compounds have been previously described as important markers for postharvest processes, sensory properties, and cultivar differentiation.^32,45^ However, their association with coffee fermentation is likely being reported for the first time. Some researchers have highlighted the lack of studies investigating how different processes, such as fermentation, influence the coffee lipidome, suggesting a need for further research in this area.^43^ A previous study reported that fatty acids are produced during coffee fermentation by yeast through extracellular hydrolysis.^50^ Another study suggested the partial conversion of palmitic acid into ethyl palmitate during the coffee fermentation process.^51^ Additionally, nuclear magnetic resonance studies have indicated a strong influence of lipids on coffee fermentation, which could explain the detection of fatty acid signals in the same spectral region.^52,53^ Furthermore, lipid oxidation produces off-flavors, and microbial degradation generates fatty acids, supporting the findings of this study. It is important to note that the fermentation studied in this paper refers to an undesired defect in coffee quality. However, various controlled fermentation mechanisms can also occur in coffee beans, leading to pleasant tastes and aromas.

Heavier species were also identified as important sensory markers in *C. arabica*, with multiple signals detected in the *m*/*z* range of 671–847. Previous research has suggested the presence of high-molecular-weight phospholipids in coffee, aligning with our findings, which exhibited low mass errors (<5 ppm). Furthermore, another study linked numerous phospholipids to key sensory properties of *C. canephora*, such as acidity, body, aromatic quality, and overall preference, further supporting our results.^54^ This study likely represents the first-time phospholipids have been directly associated with the sensory quality of *C. arabica*. Overall, phospholipids influenced all classes, with *barely soft* coffees showing a stronger presence of phosphatidylinositols, *fermented* coffees exhibiting higher levels of phosphatidic acids, and *rio* coffee presenting a unique marker at *m*/*z* 671.4639, also assigned to a phosphatidic acid.

Despite efforts to tentatively identify key compounds and their potential roles based on scientific literature, this task remains challenging. The vast diversity and abundance of chemical compounds in coffee contribute to its complex aroma, taste, and flavor profile. Further research is necessary to expand our understanding of coffee sensory science, fostering consumer-oriented innovations, improving drinkability, and enhancing the overall coffee experience.

## 4 Conclusions

In summary, the extensive information provided by LA-REIMS and high-resolution mass spectrometry in analyzing Brazilian *C. arabica* has revealed valuable sensory insights. Machine learning algorithms successfully classified coffee samples according to the Brazilian official classification, achieving up to 100% accuracy in validation set predictions. The high sensitivity observed for *barely soft*, *rio*, and *fermented* coffees suggests the presence of potential sensory markers, which were tentatively identified. For the first time, fatty acids have been directly linked to undesired fermentation in coffee, while phospholipids have been associated with the sensory quality of *C. arabica*. This study demonstrates that the LA-REIMS-based method is a viable, rapid approach for assessing the quality of large batches of *C. arabica*.

## Supporting Information Available

A figure comparing the sample and background; a table containing the training parameters for each model; a figure indicating the percentage of outliers detected for each class in the PLS-DA models; and a table detailing the figures of merit for all models(PDF)

## Abbreviations

3-CQA: 3-caffeoylquinic acid
5-CQA: 5- caffeoylquinic acid
ANN: artificial neural networks
CQAs: caffeoylquinic acids
LA-REIMS: laser-assisted rapid evaporative ionization mass spectrometry
LBFGS: limited-memory Broyden-Fletcher-Goldfarb-Shanno quasi-Newton algorithm; LV,latent variable
PLS-DA: partial least-squares discriminant analysis
SVM: support vector machine
