# Supplementary material for: Sensory Classification of Brazilian Coffea arabica by Laser-Assisted Rapid Evaporative Ionization Mass Spectrometry and Machine Learning Algorithms
Source: ACS Omega. 2025 Apr 29;10(18):18775–83. doi: 10.1021/acsomega.5c00404 (PMC12079199; doi:10.1021/acsomega.5c00404)
Supplement: Supplementary file 1 — ao5c00404_si_001.pdf [file ao5c00404_si_001.pdf]

SUPPORTING INFORMATION

Sensory classification of Brazilian *Coffea arabica* by laser-assisted rapid evaporative ionization mass spectrometry and machine learning algorithms

Victor Gustavo Kelis Cardoso[a,b], Julia Balog[c], Guilherme Post Sabin[a,d], and Leandro Wang Hantao[a,b]*

[a] Instituto de Química, Universidade Estadual de Campinas, Campinas, Brasil

[b] Instituto Nacional de Ciência e Tecnologia em Bioanalítica (INCTBio), Campinas, Brasil

[c] Waters Research Center, Budapest, Hungary

[d] OpenScience, Campinas, Brasil

* Corresponding author: Prof. Dr. Leandro Wang Hantao

Email address: wang@unicamp.br

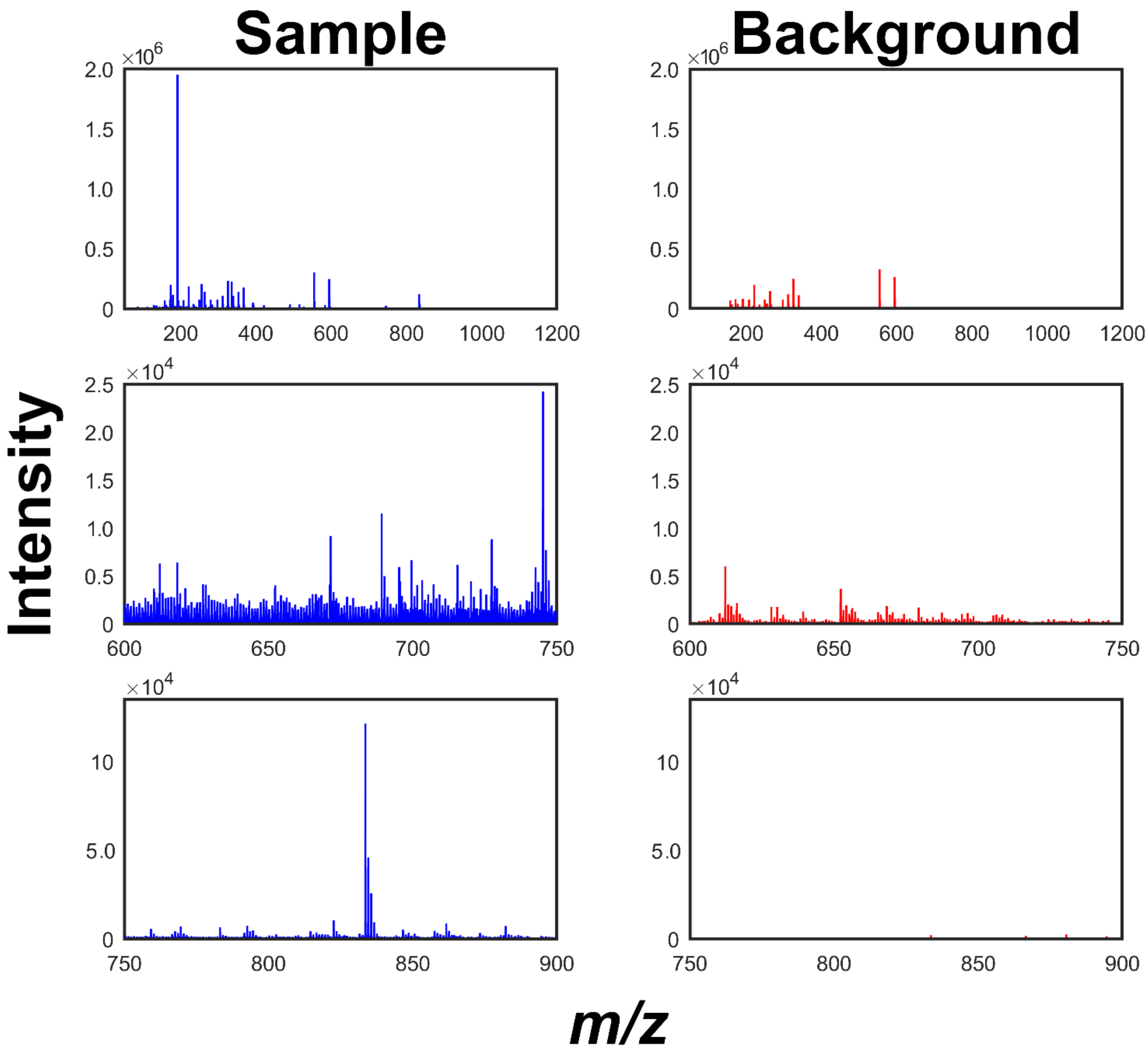

Figure S1. Comparison between sample (blue) and background (red) representative spectra across different *m*/*z* ranges.

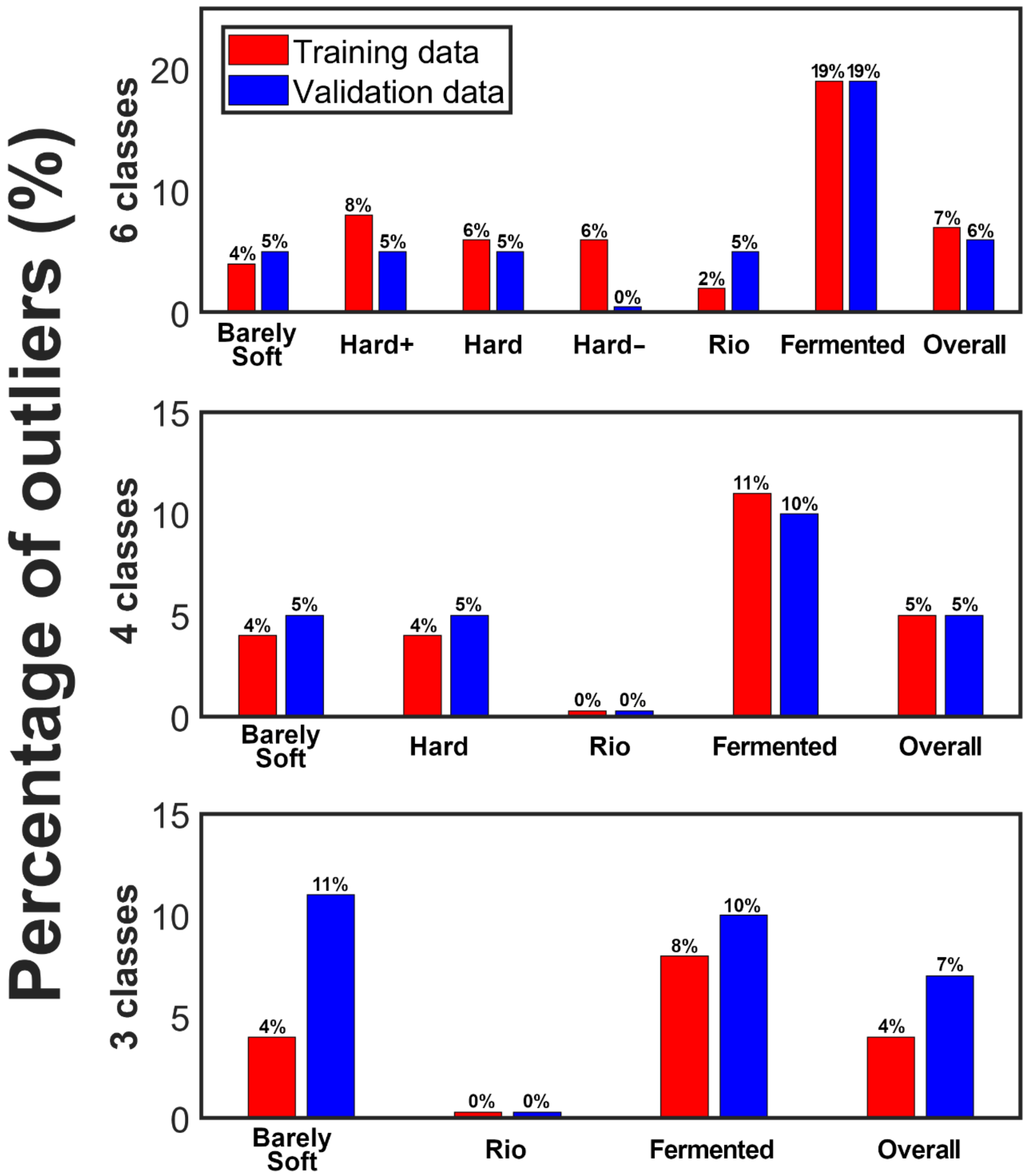

Figure S2. Percentage of outliers due to high Hoteling T^2 and Q residues identified in each class of different models in training (red) and validation (blue) sets.

Table S1. Training parameters for PLS–DA, SVM, and ANN for each group modeling.

	PLS-DA	SVM	ANN
**6 classes**	45 LV	**BS**: 0.0468; 0.0989<br>**H+**: 0.0057; 0.0468<br>**Hd**: 1.6445; 0.6452<br>**H–**: 14.5555; 0.8810<br>**Rio**: 0.0874; 0.1003<br>**Fer**: 997.3706; 6.9693	[34]<br>sigmoid activation function
**4 classes**	27 LV	**BS**: 2.5127; 0.6264<br>**Hd**: 0.0013; 0.0167<br>**Rio**: 0.0210; 0.0588<br>**Fer**: 0.0012; 0.0090	[17]<br>*tanh* activation function
**3 classes**	22 LV	**BS**: 0.0441; 0.1184<br>**Rio**: 969.4491; 11.2567<br>**Fer**: 996.8347; 9.0171	[4,104]<br>*tanh* activation function

LV: Latent variables, BS: Barely Soft, H+: Hard+, Hd: Hard, H–: Hard–, Fer: Fermented.

Note: The SVM column displays values of Box Constraint and Kernel Scale, separated by a semicolon. The ANN column contains the number of hidden layers and the corresponding number of neurons in brackets. For example, the 3-classes model includes two hidden layers, with the first containing 4 and the second containing 104 neurons.

Table S2. Figures of merit for PLS–DA, SVM and ANN models in predicting training and validation sets based on the different modelled groups.

		Training set			Validation set		
		**PLS-DA**	**SVM**	**ANN**	**PLS-DA**	**SVM**	**ANN**
**6 classes**	Acc	83%	91%	100%	61%	67%	70%
	Sn	83%	91%	100%	61%	67%	70%
	Pr	83%	91%	100%	63%	65%	70%
**4 classes**	Acc	85%	94%	100%	77%	83%	85%
	Sn	85%	94%	100%	77%	83%	85%
	Pr	85%	95%	100%	76%	82%	85%
**3 classes**	Acc	90%	97%	99%	92%	100%	97%
	Sn	90%	97%	99%	92%	100%	97%
	Pr	91%	97%	99%	92%	100%	97%

Acc: accuracy, Sn: average sensitivity, and Pr: average precision.

PLS-DA — TRAINING

Predicted class \ Actual class	BS	H+	HARD	H-	RIO	FER
BS	98	4	2	2	2	1
H+	2	92	3	3	1	4
HD	1	4	94	2	0	1
H-	2	4	4	101	4	2
RIO	2	2	6	2	107	1
FER	1	3	3	1	1	86
OUT	4	9	7	7	2	23

PLS-DA — VALIDATION

Predicted class \ Actual class	BS	H+	HARD	H-	RIO	FER
BS	14	1	0	2	0	0
H+	0	9	2	3	1	0
HD	3	5	12	2	3	2
H-	1	4	2	10	0	0
RIO	0	0	4	2	16	0
FER	0	1	0	2	0	15
OUT	1	1	1	0	1	4

SVM — TRAINING

Predicted class \ Actual class	BS	H+	HARD	H-	RIO	FER
BS	102	5	7	2	2	1
H+	1	98	3	2	0	1
HD	1	4	100	2	1	1
H-	3	7	1	110	2	1
RIO	2	2	6	2	112	1
FER	0	2	2	1	0	113

SVM — VALIDATION

Predicted class \ Actual class	BS	H+	HARD	H-	RIO	FER
BS	16	2	2	2	0	0
H+	0	7	3	2	0	0
HD	2	5	10	0	1	1
H-	1	5	3	10	0	0
RIO	0	0	3	4	20	0
FER	0	2	0	3	0	20

ANN — TRAINING

Predicted class \ Actual class	BS	H+	HARD	H-	RIO	FER
BS	110	0	0	0	0	0
H+	0	118	0	0	0	0
HD	0	0	119	0	0	0
H-	0	0	0	118	0	0
RIO	0	0	0	0	117	0
FER	0	0	0	0	0	118

ANN — VALIDATION

Predicted class \ Actual class	BS	H+	HARD	H-	RIO	FER
BS	17	1	2	4	0	0
H+	1	11	2	2	1	1
HD	1	4	12	1	3	1
H-	0	4	2	11	0	0
RIO	0	0	3	1	17	0
FER	0	1	0	2	0	19

Figure S3. Confusion chart for PLS-DA, SVM, and ANN models while modeling 6 sensory quality classes for training and validation sets. BS: *barely soft*, H+: *hard+*, HD: *hard*, H-: *hard-*, FER: *fermented*, OUT: *outlier*.

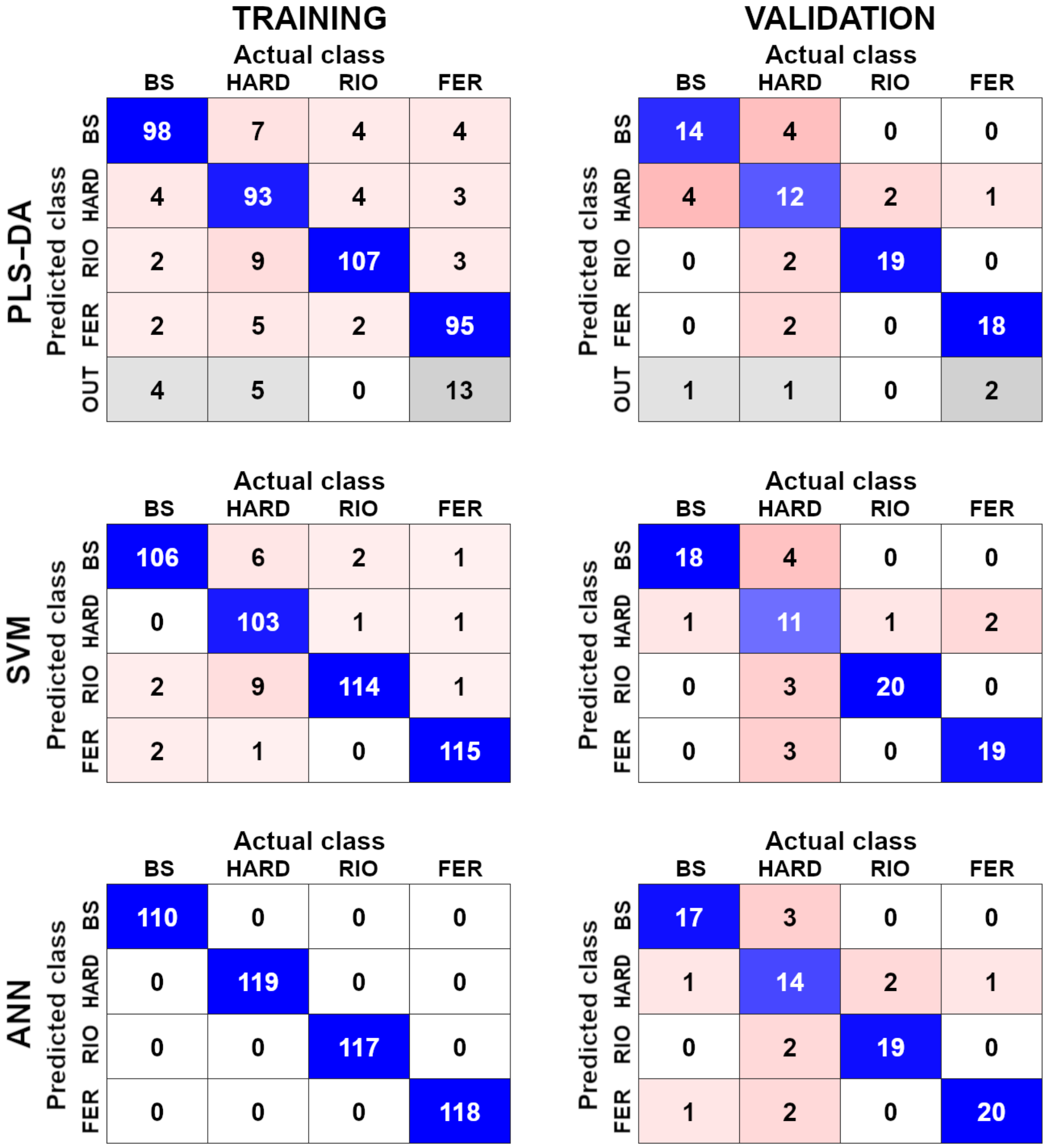

Figure S4. Confusion chart for PLS-DA, SVM, and ANN models while modeling 4 sensory quality classes for training and validation sets. BS: *barely soft*, FER: *fermented*, OUT: *outlier*.

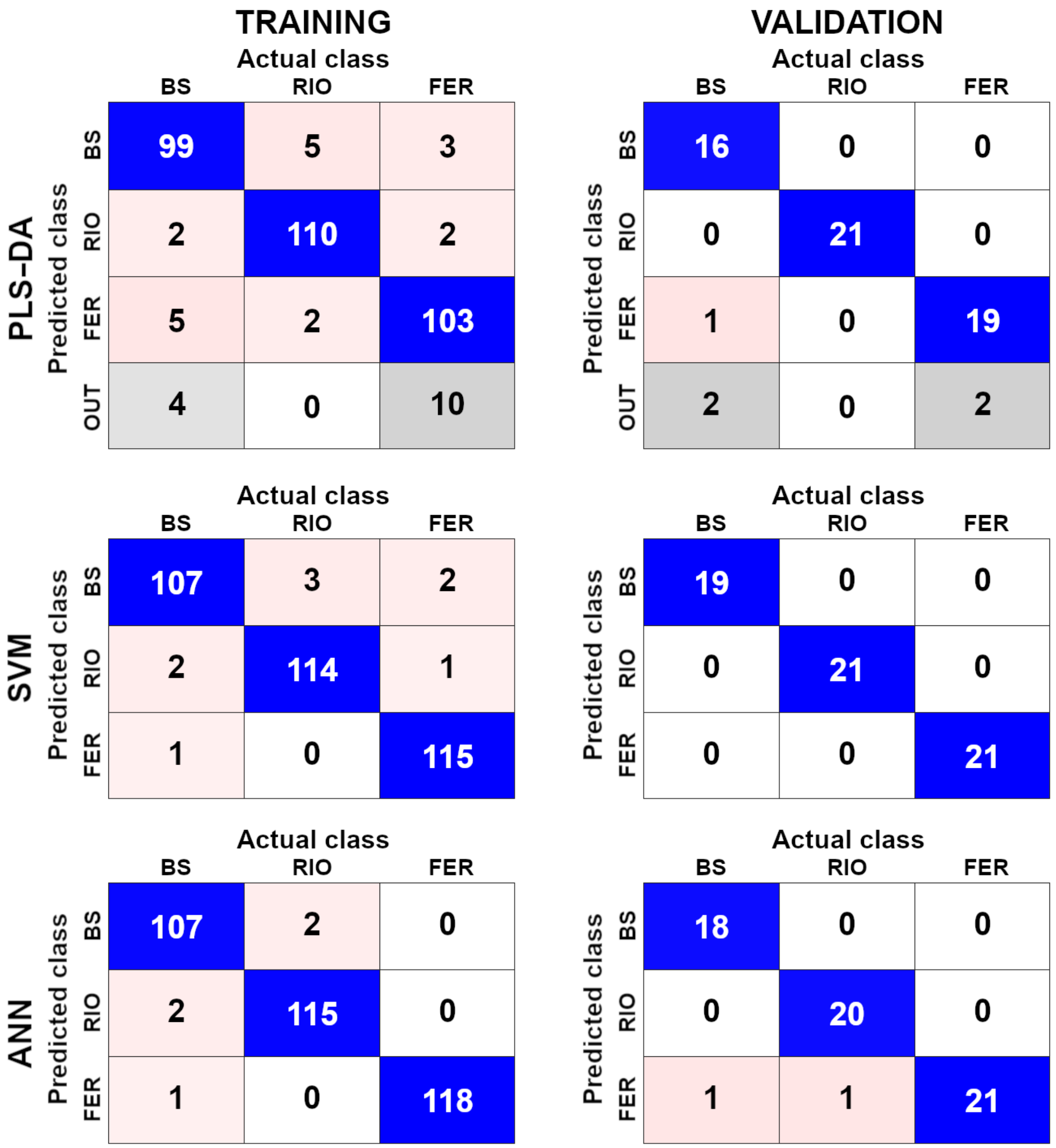

Figure S5. Confusion chart for PLS-DA, SVM, and ANN models while modeling 3 sensory quality classes for training and validation sets. BS: *barely soft*, FER: *fermented*, OUT: *outlier*.
